# Antiviral Activity of Flavonoids from Geopropolis of the Brazilian Jandaira Bee against Zika and Dengue Viruses

**DOI:** 10.3390/pharmaceutics15102494

**Published:** 2023-10-19

**Authors:** Poliana Gomes da Silva, Elton José Ferreira Chaves, Tania Maria Sarmento Silva, Gerd Bruno Rocha, Willyenne Marília Dantas, Ronaldo Nascimento de Oliveira, Lindomar José Pena

**Affiliations:** 1 Laboratory of Virology and Experimental Therapy (Lavite), Department of Virology, Aggeu Magalhães Institute (IAM), Oswaldo Cruz Foundation (Fiocruz), Recife 50670-420, Pernambuco, Brazil; poligs250@gmail.com (P.G.d.S.); chavesejf@cbiotec.ufpb.br (E.J.F.C.); dantaswillyenne@gmail.com (W.M.D.); 2 Phytochemical Bioprospecting Laboratory, Department of Chemistry, Federal Rural University of Pernambuco, Recife 52171-900, Pernambuco, Brazil; sarmentosilva@gmail.com; 3 Laboratory of Computational Quantum Chemistry, Department of Chemistry, Federal University of Paraiba, João Pessoa 58050-085, Paraiba, Brazil; gbr@quimica.ufpb.br; 4 Bioactive Compounds Synthesis Laboratory, Department of Chemistry, Federal Rural University of Pernambuco (UFRPE), Recife 52171-900, Pernambuco, Brazil; ronaldon38@gmail.com

**Keywords:** Zika virus, dengue, antivirals, naringenin, 7-*O*-methyl naringenin, geopropolis, molecular simulations

## Abstract

Arthropod-borne viruses within the *Flaviviridae* family such as Zika (ZIKV) and dengue (DENV) are responsible for major outbreaks in tropical countries, and there are no specific treatments against them. Naringenin and 7-*O*-methyl naringenin are flavonoids that can be extracted from geopropolis, a natural material that the Brazilian Jandaira stingless bee (Melipona subnitida Ducke) produces to protect its nest. Here, these flavonoids were tested against ZIKV and DENV using Vero cells as a cellular model to perform a cytotoxicity assay and to define the effective concentrations of TCID_50_ as the readout method. The results demonstrated the antiviral activity of the compounds against both viruses upon the treatment of infected cells. The tested flavonoids had antiviral activity comparable with 6-methylmercaptopurine riboside (6-MMPr), used here as a positive control. In addition, to identify the possible action mechanism of the antiviral candidates, we carried out a docking analysis followed by a molecular dynamics simulation to elucidate naringenin and 7-*O*-methyl naringenin binding sites to each virus. Altogether, these results demonstrate that both flavonoids have potent antiviral effects against both viruses and warrant further *in vivo* trials.

## 1. Introduction

The antiviral activity of flavonoids is well reported for a variety of viruses, including the members of the *Flaviviridae* family [1,2]. More specifically, the antiviral activity of naringenin against ZIKV and DENV has been reported before [3,4], but these studies have not been independently reproduced, and there is still a lack of update data. In addition, there are no studies on the antiviral action of naringenin or its derivative 7-*O*-methyl naringenin against these viruses using these compounds in the context of bee-derived products. ZIKV and DENV belong to the same genus (*Flavivirus*) and share molecular, structural and epidemiological similarities. These viruses have become endemic in the tropical and subtropical zones of the globe, causing a significant burden to the health systems of these countries. In 2020, we warned about the imminent risk of arbovirus epidemics in Brazil due to the mobilization of human resources and diagnostic laboratories that were historically dedicated to arbovirus control to fight the COVID-pandemic [5]. In fact, Brazil is facing a large dengue outbreak, and ZIKV can reemerge at epidemic levels any time. According to the most recent epidemiological report, from January 2023, Brazil registered 1,450,270 probable cases of dengue fever (rate of incidence of 679.9 cases per 100,000 inhab.) and 9204 probable cases of Zika, corresponding to an incidence rate of 4.3 cases per 100 thousand inhabitants [6].

ZIKV causes a self-limiting exanthemic disease in most patients [7]. However, the virus is neurotropic, and its infection can result in the development of severe neurological disorders in adults and in newborns [8,9]. In 2015, Brazil reported for the first time an accumulation of microcephaly cases and other teratogenic defects in newborn babies associated with the ZIKV infection. The causal relationship was later categorically confirmed, and the virus became an important issue to the world public health scenario [10]. In February 2016, Zika-virus-induced disease was classified by the World Health Organization (WHO) as a Public Health Emergency of International Concern (PHEIC) [11]. Currently, the virus is endemic in almost 80 countries [8]. In the recent past, there was a significant outbreak in India, as reported in the Zika epidemiology update of February 2022 [12]. As a consequence, the virus remains a global concern pathogen with continuous growth in cases [13]. Despite this, there are neither effective vaccines nor approved drug therapies for Zika, making the development of antivirals a priority for Zika-endemic countries.

Related to DENV at present, the virus has four antigenically distinct serotypes: DENV-1, DENV-2, DENV-3 and DENV-4, and all result in similar clinical manifestations. The clinical manifestations caused by DENV-2 infection result in a severe disease outcome when compared to the other serotypes [9,14,15]. DENV-2 serotype was present in the main dengue outbreaks since 1990, when it was first documented [15,16]. Based on data made available by the WHO in the Americas, Asia and the Western Pacific, the incidence of DENV increased by 30 times in the last 50 years. It is estimated that up to 400 million infections occur annually in more than 152 endemic countries, putting almost half of the world’s population at risk [17,18,19]. The disease is still a great concern among issues related to global health, given that approximately 100 million people are at risk of infection each year [10,18]. Dengue outbreaks are repeatedly reported in almost all continents [20,21,22,23], which demonstrates how critical this situation remains. There are many ongoing studies to find effective antiviral drugs to inhibit the virus mechanisms [24]. In addition, not too many years ago, a live-attenuated chimeric yellow fever/tetravalent dengue vaccine (CYD-TDV), Dengvaxia, was licensed by the Food and Drug Administration (FDA) for disease prevention, but the vaccine is still in clinical trials and not yet available to the population [25,26]. Furthermore, its therapeutic approach provides only partial cross-protection for the four serotypes of DENV [27].

One possible source of compounds to fight these viruses can be bioprospected from the geopropolis of certain bee species. Bees from the subfamily Meliponinae (Hymenoptera: Apidae), known as “stingless bees”, are pollinating insects, distributed through the tropical regions of the planet, especially in South America [28]. Among the various members of this subfamily, the Jandaira bee (Melipona subnitida Ducke) is an endemic species that naturally occurs in an arid area located in Northeastern Brazil named caatinga [29]. This bee species uses various materials for the construction and protection of their nests against enemies, such as resins, wax, clay and soil. As a result, the mixture is a characteristic colored clay with a hard consistency [28,29]. The traditional propolis was then named with the prefix geo, in relation to the Greek word geo (the Earth, in its broadest meaning), becoming defined as geopropolis [28]. Antioxidant and antibacterial activities of geopropolis have already been described [30]. In addition, previous studies have identified the antiviral action against herpes simplex type 1 (HSV-1) by the hydromethanolic extract of geopropolis from Scaptotrigona postica [31]. However, antiviral activity against other viruses of interest to public health, especially RNA viruses, has not yet been evaluated.

## 2. Materials and Methods

### 2.1. Materials

Geopropolis samples were collected from Jandaira bees (*Melipona subnitida* Ducke) at Sítio Riacho, located in Vieirópolis, a city from the semi-arid region of Paraiba state, Brazil. Thiopurine nucleoside analogue (6-MMPr) was obtained from Sigma-Aldrich (Saint Louis, MI, USA). Vero cells were obtained from the Fiocruz Pernambuco Laboratory of Virology repository. All studies were conducted with the ZIKV strain named ZIKV/H. sapiens/Brazil/PE243/2015 (GenBank: KX197192.1), which was isolated in the State of Pernambuco, Brazil from patient with an exanthematous disease. The 3808/BR-PE/95 strain of DENV-2 (GenBank: EU259569) was isolated from a dengue case in 1995 in Pernambuco state. 

### 2.2. Geopropolis Sample and Flavonoid Isolation Process

Geopropolis samples (550.0 g) were extracted using an EtOH ultrasound bath (for 20 times during 15 min each time ). In sequence, using a rotary evaporator at 40 °C, the extract was then filtered and concentrated to obtain a furnish ethanolic extract (25.8 g) (Figure S1). A portion of EtOH extract (15.7 g) was dissolved in MeOH: H_2_O (1:1) (200 mL), maintained in sonication for 30 min. The obtained solution was subjected to successive extractions with hexane (5 × 150 mL) and ethyl acetate (5 × 100 mL). The solvents were removed using a rotavapor, obtaining hexane (4.8 g), ethyl acetate (4.5 g) and MeOH: H_2_O (0.7 g) as fractions. The resulting EtOAc, fraction, rich in flavonoids, was then submitted to isolation, and the flavonoids naringenin and 7-*O*-methyl naringenin were identified [30] (Figure S1A,B).

### 2.3. Cells and Viruses

Vero cells’ monolayers were cultivated using Dulbecco’s modified Eagle’s medium (DMEM) (Gibco, Carlsbad, CA, USA). The medium solution was supplemented with 10% inactivated fetal bovine serum (FBS) (Gibco), 2 mM l-glutamine (Gibco) and 100 U/mL penicillin/streptomycin (Gibco). Both ZIKV and DENV-2 were amplified and titrated in this cell line using the same media, but with 2% FBS.

### 2.4. Cell Viability Assay

The cell toxicity induced by the three tested fractions and the isolated flavonoids was evaluated in a growing Vero cell. The mitochondrial reduction reaction of the tetrazolium dye 3-(4,5-dimethylthiazol-2-yl)-2,5-diphenyltetrazolium bromide (MTT) (Sigma) was used to define the cell viability. To this end, Vero cells (1 × 10^4^ cells/well) were plated in 96-well microplates 24 h beforehand. The cells received an increasing range of concentrations (12.5 to 250 µM/mL for naringenin and 50 to 200 µM/mL for 7-*O*-methyl naringenin) of the test compounds diluted in dimethyl sulfoxide (DMSO). The final concentration of DMSO did not induce a significantly cytotoxic effect on Vero cells, since its concentration value was equal to or lower than 0.06% *v*/*v*. After a period of 120 h at 37 °C in a 5% CO_2_ atmosphere, the culture medium was removed from the plate, and 50 μL of recently prepared MTT solution (1 mg/mL) was then added. The 96-well microplate was placed in a CO_2_ incubator for 4 h at 37 °C. After this period, the MTT solution was replaced by DMSO. Then, the optical density at 540 nm (OD540) was measured using a BioTekTM ELx800TM 96-well plate reader (BioTek Instruments Inc., Winooski, VT. The OD540 subtraction of treated cells compared to untreated cells was calculated. With this information, the cytotoxic concentration for 50% of the cell culture (CC_50_, which means the concentration of compound related to a 50% reduction in cell viability) and the CC_20_ (the concentration that caused only a 20% reduction in cell viability) were defined. The CC_20_ value was empirically defined as the maximum safe limit to perform the antiviral screening [32]. Results were expressed as mean ± SD of three independent replicates.

### 2.5. Antiviral Assay

One day prior to infection with the ZIKV or DENV strain, Vero cells were seeded in 24-well plates (5 × 10^4^ cells/well). The infection was performed at the multiplicity of infection (MOI) of 0.1 for 2 h at 37 °C in 5% CO_2_. After virus internalization, the inoculum was removed, and the cells were washed using the growth medium. In sequence, the supernatant was then replaced with fresh medium containing AcOEt subfractions, naringenin or 7-*O*-methyl naringenin at their CC_20_ concentrations as determined in the cell viability assay. 6-MMPr, used as positive control drug, was also included as a drug treatment in some wells [33]. Controls of mock (non-infected) and infected non-treated cells were included. At 120 h post-infection (hpi), the cytopathic effect was evaluated using an inverted microscope (AE2000 binocular microscope, Motic, Hong Kong), and cells were photographed with a smartphone. The cell supernatant was harvested at 120 hpi and stored at −80 °C for posterior virus titration, using the TCID_50_ method.

### 2.6. Viral Titration

The TCID_50_ method was used here to perform viral titration [34]. The titer values were expressed as log_10_ TCID_50_/mL. One day prior to titration, Vero cells were seeded in a 96-well plate (1 × 10^4^ cells/well) at 37 °C in a 5% CO_2_ incubator. The cell supernatants that were previous stored (see item 2.5—cytopathic effect assay) were serially diluted 10-fold in DMEM and submitted to the cell monolayer. After incubation for 5 days at 37 °C and 5% CO_2_, the cytopathic effect was observed through an inverted optical microscope, and the reduction in viral titer was defined according to Reed–Muench parameters [34].

### 2.7. Statistical Analysis

The differences in viral titer of treated and untreated infected cells were analyzed according to ANOVA followed by the Dunnett test using GraphPad Prism software v.5.01 (GraphPad Software, La Jolla, CA, USA). The drug concentration that reduced viral titer by 50% (IC_50_) was defined using the same software. This represents the compound concentration required to reduce ZIKV or DENV titer by 50% when compared with the non-treated cells. The CC_50_ and CC_20_ values were also calculated using GraphPad Prism software in a non-linear regression interpretation. The selective index (SI) was based on the ratio math of CC_50_ and IC_50_ values. Data were reported as the mean ± standard deviation (SD) of three independent experiments. Significance was only considered when *p* < 0.05.

## 3. Theoretical Methods

### 3.1. Molecular Docking

A semiflexible molecular docking approach was performed to predict the binding affinity of naringenin and 7-*O*-methyl-naringenin at the binding sites of the most important nonstructural proteins of DENV2 and ZIKV: (i) NS3-helicase; (ii) NS3-protease; (iii) NS5-methyltransferase; and (iv) NS5-RdRp. The X-ray structures used in docking simulations were downloaded from the Protein Data Bank; the accession codes are shown in Table 1.

The water and ion molecules were properly removed from the original X-ray structure, and the protonation state of titratable residues at physiological pH was evaluated on the PDB2PQR platform [43]. The remaining structures were aligned (with similar structures), and the center of mass of the inhibitor/substrate was calculated and used to position the grid box in the docking simulations. The 3D coordinates of naringenin and 7-*O*-methyl-naringenin were obtained from the PubChem platform. The partial charge in the atoms was estimated considering the RESP method [44] using Gaussian software and the AMBERTOOLS package. Then, receptors and ligands were properly prepared using the scripts prepare_receptor4.py and prepare_ligands4.py, both of which are available in the MGLTools package. Docking simulations were performed using the AutoDock Vina software (version 1.1.2) [45] set with exhaustiveness equal to 32; other parameters were kept in default mode.

### 3.2. Enthalpy of Binding

The binding poses obtained in the molecular docking step (Section 3.1) were rescored using a semiempirical quantum method in order to estimate the enthalpy of binding. MOPAC2016 software [46] was used, and all calculations were performed considering the following parameters: (i) PM7 semiempirical Hamiltonian; (ii) linear scaling algorithm MOZYME with a cutoff radius of 9 Å; (iii) SCF convergence criteria in the default configuration; (iv) COSMO implicit solvent field with a relative permittivity of 78.4; and (v) an effective solvent molecule radius of 1.3 Å. The enthalpy of binding was calculated using Equation (1):

(1) $${∆H}_{bind}={∆H}_{f}^{complex}-\left({∆H}_{f}^{receptor}+{∆H}_{f}^{ligand}\right)$$

### 3.3. Molecular Dynamics Simulation

A molecular dynamics (MD) simulation was performed using NAMD [47] software (version 2.13). The protein and ligand were parameterized using the FF19SB [48] and GAFF2 force fields, respectively. In this step, we did not recalculate the partial charges of the ligand. In other words, the RESP charges [44] calculated in the previous step were kept for the molecular dynamics step as well. Furthermore, MD simulation was performed with the following parameters: (i) periodic boundary conditions; (ii) restriction of vibration for covalent bonds involving hydrogen atoms, HOH angles and the OH bond distance of TIP3P water molecules (SHAKE algorithm); (iii) time step equal to 2 fs; (iv) electrostatic interaction cutoff of 12 Å for all steps of the simulations; and (v) the Particle Mesh Ewald (PME) method was used for long-range electrostatic interaction. The starting geometry was submitted to minimization, heating from 0.0 to 310.0 K and pressurization steps. Finally, a pressure of 1.0 atm was kept with the Langevin piston barostat, and the system was simulated for 50 ns without restraints; frames were captured every 5 ps.

### 3.4. Interaction Energy Analysis

There are several methodologies capable of quantifying protein–ligand interactions from a large number of frames of the MD simulation; however, we used the MM/PBSA method [49,50]. The Molecular Mechanics Poisson–Boltzmann Surface Area (MM/PBSA) methodology has been extensively used as a proficient and dependable method for free energy simulations, specifically for modeling molecular recognition phenomena such as protein–ligand binding interactions [49]. The interaction energy profile between the protein and ligand was calculated using the MMPBSA.py [51] script available in the AMBERTOOLS software suite. The following parameters were used: (i) 200 frames of the last 25 ns of the equilibrium MD simulation; (ii) implicit solvent; and (iii) 0.15 M salt concentration (NaCl).

## 4. Results

### 4.1. Identification of Flavonoids

**Figure 1:**
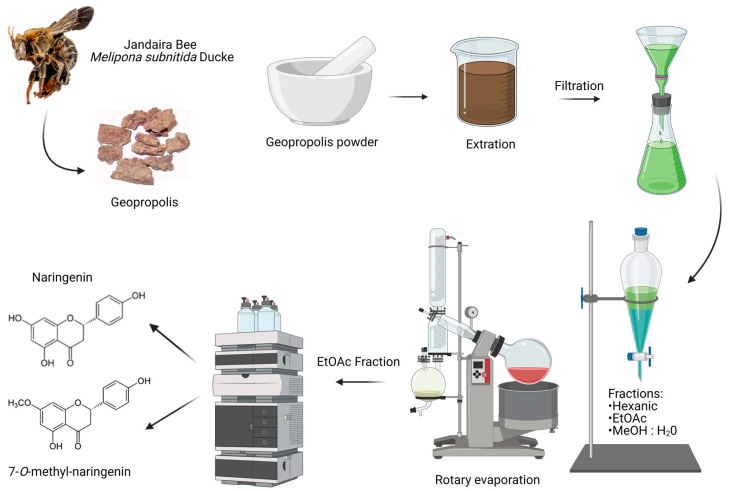
Naringenin and 7-*O*-methyl naringenin isolation process. Created with BioRender.com—agreement number: SN25BZXSSA.

The detailed procedures on the obtainment of extracts, fractions and isolated flavonoids naringenin and 7-*O*-methyl naringenin have been previously published by our group [52,53,54]. A schematic figure is used here to illustrate the compound obtention process in Figure 1.

### 4.2. Flavonoids’ Citotoxic Effects

**Figure 2:**
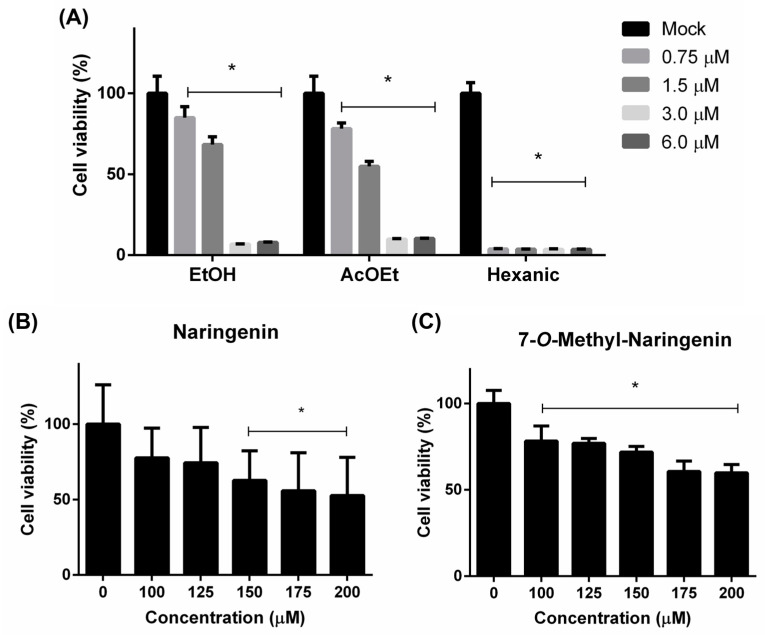
Cytotoxicity to Vero cells of the ethanolic extract (EtOH), ethyl acetate (AcOEt) and hexane fractions (**A**) and the isolated compounds 7-*O-*methyl naringenin (**B**) and naringenin (**C**). The cell monolayer was either treated or not treated (Mock) with a range of concentrations of each sample and incubated for 120 h. Then, cell viability was assessed trough the MTT method. Concentration values are described in µM/mL. Results are expressed as mean ± SD of 3 independent experiments. * *p* < 0.05.

The cytotoxicity of fractions and isolated compounds was tested on Vero cells using the MTT method. The hexane fraction was demonstrated to be the most cytotoxic, whereas AcEOt and EtOH tended to be less toxic to Vero cells (Figure 2A). These primary results led to the following AcEOt subfraction preparations, where naringenin and 7-*O*-methyl naringenin (Figure 2B,C) were identified, isolated and then tested against both viruses.

The CC_50_ and CC_20_ values of each tested substance are described on Table 2. All concentrations are expressed in µM/mL to easily compare each sample. The maximal non-toxic concentration is defined by the CC_20_ values resulting from the antiviral assay.

### 4.3. Post-Treatment Antiviral Activity of Flavonoids

**Figure 3:**
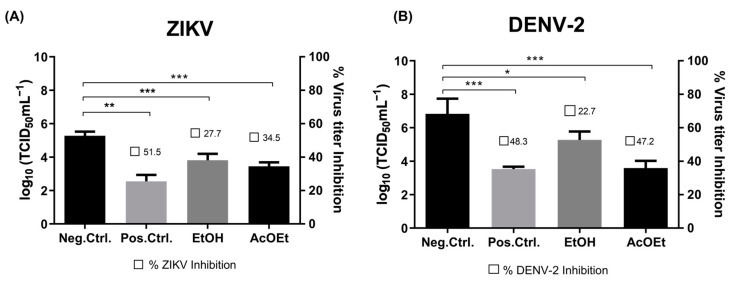
Effect of ethanolic extract (EtOH) and ethyl acetate (AcOEt) fractions on the virus replication process. Vero cells were infected (MOI: 0.1) with either ZIKV (**A**) or DENV (**B**) for 2 h. After that, the cells were treated with CC_20_ of each sample and incubated for 120 h. In sequence, the supernatant was collected and submitted to TCID_50_ measurement. Non-treatment wells (DMEM only) are defined as negative control—Mock, and 6-MMPr as a positive control drug. µM/mL describes the values of concentration. Three independent experiments expressed mean ± SD. * *p* < 0.05, ** *p* < 0.01, *** *p* < 0.001.

The antiviral effect of the geopropolis fractions and isolated compounds against ZIKV and DENV was tested. The cell monolayer in each well was infected with ZIKV or DENV (MOI of 0.1) for 2 h at 37 °C in 5% CO_2_. Then, the previous inoculum was removed and fresh medium containing the test fraction or compound, at their CC_20_ concentration, was added to the cells. At different times post-infection, the cell monolayer was examined, and the supernatant was titrated by TCID_50_ to measure virus reduction. Figure 3 demonstrates the effects of the ethanolic extract (EtOH) and ethyl acetate (AcOEt) fractions on the virus titer.

**Figure 4:**
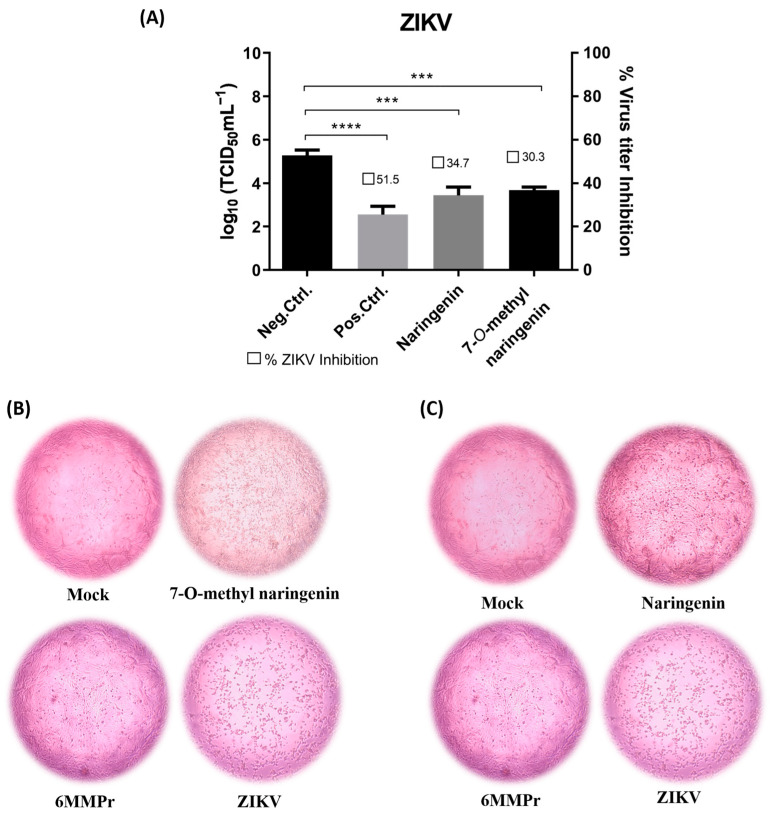
(**A**): Effect of naringenin and 7-*O*-methyl naringenin on virus replication. Vero cells were infected with ZIKV at a MOI of 0.1 for 2 h. The cells were treated with CC_20_ concentration of each tested sample for 120 h. The supernatant of each well was collected, and its titter was defined using TCID_50_. The DMEM medium was used here as a negative control (non-treatment), and 6-MMPr was used as a positive control drug. µM/mL describes the values of concentration. Three independent experiments expressed mean ± SD. *** *p* < 0.001, **** *p* < 0.0001. (**B**,**C**): Vero cell monolayers presenting ZIKV-induced cytopathic effect after naringenin and 7-*O*-methyl naringenin treatment. Non-treatment wells (DMEM only) are defined as negative control—Mock, and 6-MMPr as a positive control drug.

**Figure 5:**
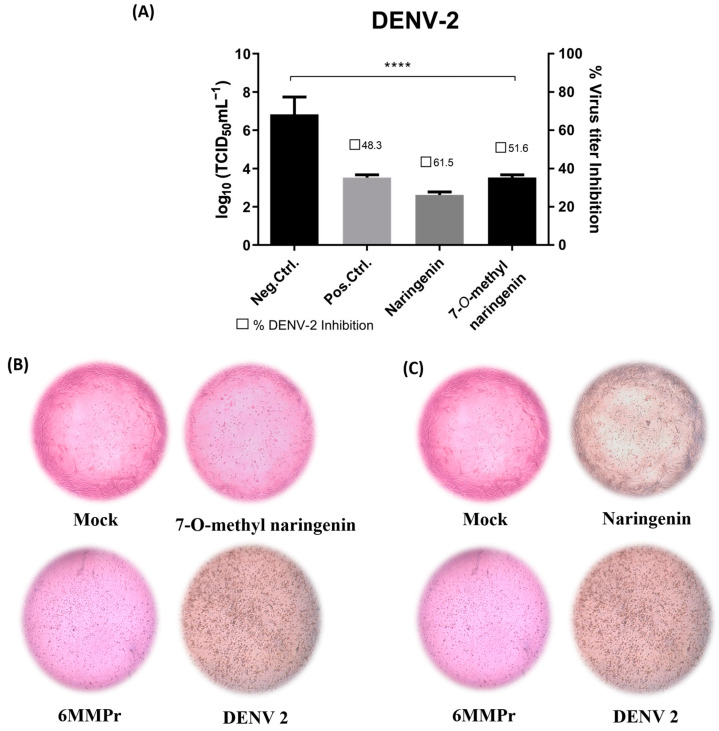
(**A**): Effect of naringenin and 7-*O*-methyl naringenin on virus replication. Vero cells were infected with DENV at a MOI of 0.1 for 2 h. The cells were treated with CC_20_ concentration of each tested sample for 120 h. The supernatant of each well was collected, and its titter was defined using TCID_50_. The non-treatment with DMEM medium is here used as a negative control, and 6-MMPr was used as a positive control drug. µM/mL describes the values of concentration. Three independent experiments expressed mean ± SD. **** *p* < 0.0001. (**B**,**C**): DENV-induced cytopathic effect after naringenin and 7-*O*-methyl naringenin treatment in Vero cells. Non-treatment wells (DMEM only) are defined as negative control—Mock, and 6-MMPr as a positive control drug.

Both naringenin and 7-*O*-methyl naringenin flavonoids significantly (*p* < 0.05) reduced the viral titer when incubated with Vero cells for 120 h post-infection (Figure 4A and Figure 5A). Naringenin at 86.43 µM/mL and 7-*O*-methyl naringenin at 105.2 µM/mL (corresponding to their CC_20_) were able to decrease ZIKV viral titer by 40 to 60%, respectively, whereas DENV titer was reduced by 60 to 80% using the same concentrations of the drugs. At 60.5 µM/mL, 6-MMPr used as a positive control and also inhibited both viruses in a percentage range of 60–80%.

Figure 4B,C as well as Figure 5B,C present the effect of naringenin and 7-*O*-methyl naringenin in protecting Vero cells against ZIKV and DENV infections, respectively. Both substances prevented morphological modifications usually associated with the infection progress. Characteristic features, such as shrinkage and clumping, were avoided by the drug presence, and the preservation of cell monolayer integrity was demonstrated (see “ZIKV” and “DENV” subtitled images).

Table 3 and Table 4 summarize the cytotoxicity and antiviral endpoints calculated for naringenin, 7-*O*-methyl naringenin and 6-MMPr against ZIKV and DENV, respectively. For ZIKV, 7-*O*-methyl naringenin had the highest SI (18.7), which indicates that it is 18.7 times more toxic for the virus than for the cells. The SI of naringenin and 6-MMPr was 4.1 and 11.9, respectively. For DENV, the test compounds naringenin and 7-*O*-methyl naringenin had a SI of 5.6 and 7.7, respectively, which was lower than that of 6-MMPr (SI = 16.0)

### 4.4. The Flavonoids Naringenin and 7-O-Methyl Naringenin Bind to the NS5-Methyltransferase of Both DENV and ZIKV

**Figure 6:**
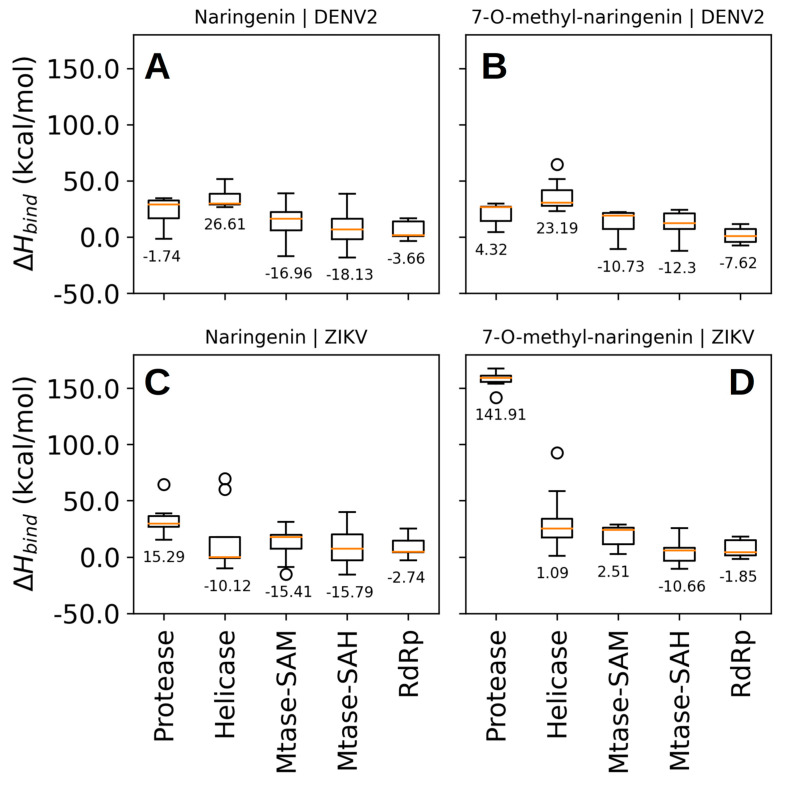
Rescore of docking poses. Binding enthalpy calculated by a semiempirical quantum method was used to rank the docking poses (total of 9 poses per target) predicted by Vina software. Naringenin binding interaction with DENV-2 (**A**) and ZIKV (**C**) nonstructural proteins; 7-O-metlyl-naringenin binding interaction with DENV-2 (**B**) and ZIKV (**D**) nonstructural proteins.

**Figure 7:**
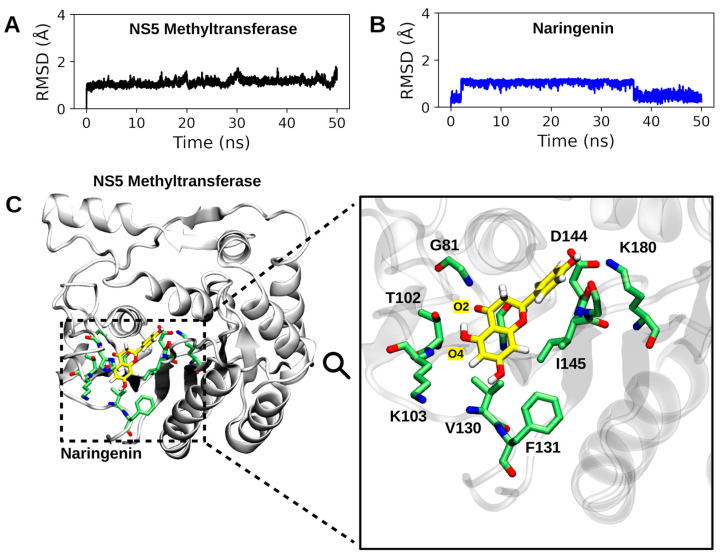
Summary of MD simulation results. In (**A**,**B**), the RMSD profiles of the NS5-methyltransferase:naringenin complex are presented. In (**A**), the RMSD profile of the NS5 subunit (backbone atoms) is shown, and in (**B**), the RMSD profile of the heavy atoms of naringenin. (**C**). Binding pose of naringenin at the binding site of NS5-methyltransferase. The entire complex is shown on the left, and the active site residues make close contact (<4.5 Å) with naringenin. The hydrogen atoms of the protein residues have been omitted to improve visualization. The carbon atoms of naringenin are shown in yellow.

The binding poses predicted by Vina software showed relative binding affinities be-tween −6.6 and −8.3 kcal/mol (Figure S2). Considering these scores, we were not able to discern with confidence to which molecular target both compounds can bind in a high affinity profile (Figure S2). Therefore, we rescored the predicted binding poses using the enthalpy of binding calculated by the PM7 semiempirical quantum chemical method. This time, we found binding poses with energies between −18 and 142 kcal/mol and concluded that naringenin binds to NS5-methyltransferase of DENV-2 with higher affinity, ΔHbind = −18.13 kcal/mol (Figure 6). In contrast to the binding affinities calculated by Vina software, the calculated binding enthalpies indicated that both compounds (naringenin and 7-*O*-methyl-naringenin) interact weakly at the binding site of the protease and helicase domains of the NS3 protein of DENV-2. For the RdRp domain of the NS5 protein, we observed binding poses with binding enthalpies between −3.66 and −7.62 kcal/mol (Figure 7A,B). This means a lower affinity when compared to the binding enthalpy observed for NS5-methyltransferase. Regarding the predicted poses for the NSPs of ZIKV, we were able to identify that naringenin weakly interacts with the protease domain of the NS3 protein and the RdRp domain of the NS5 protein (Figure 6C). We observed that naringenin interacts at the helicase domain binding site with a binding enthalpy of −10.12 kcal/mol, a high affinity, but not larger than that calculated for naringenin when bound to the NS5-methyltransferase binding site of DENV-2. We found similar results when naringenin and 7-*O*-methyl-naringenin bind to the NS5-methyltransferase binding site of ZIKV (Figure 7A,C,D). Finally, 7-*O*-methyl-naringenin interacts weakly at the binding site of the protease domain of the NS3 protein and the RdRp domain of NS5 (Figure 7D). Since naringenin has a high affinity for the NS5-methyltransferase of DENV2, we performed a molecular dynamics simulation to evaluate the stability of this compound at the NS5 binding site and evaluated the key intermolecular interactions between them.

### 4.5. Molecular Dynamics Simulation Showed That Naringenin Forms a High Number of Non-Covalent Interactions at the Binding Site of NS5-Methyltransferase

**Figure 8:**
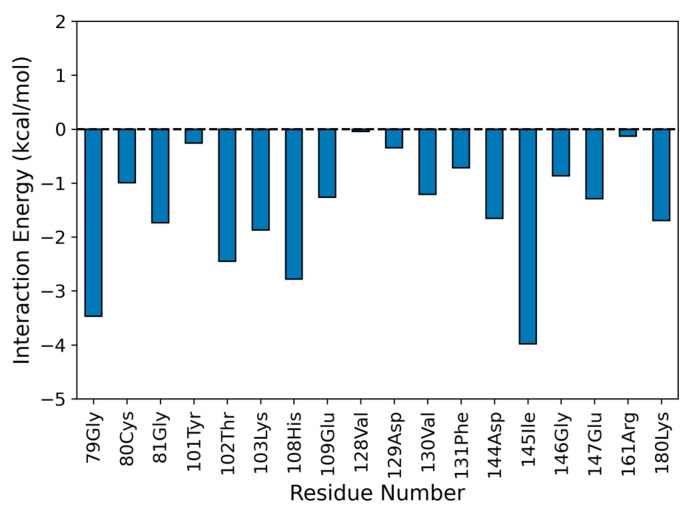
Interaction energy profile between naringenin and the binding site of NS5-methyltransferase. The interaction energy was calculated by considering 200 frames of the last 25 ns of the MD simulation. An interaction energy > 0 comprises a repulsive interaction, whereas an interaction energy < 0 is attractive.

Considering the time interval of 50 ns, we observed that the NS5 subunit and naringenin do not undergo abrupt changes in their conformation (average RMSD of 1.109 ± 0.134 Å for NS5-methyl-transferase and 0.872 ± 0.303 Å for naringenin; see Figure S1). Figure 7 shows the binding pose of naringenin at the NS5-methyltransferase binding site (last frame of the MD simulation). The analysis of noncovalent interactions pointed to the occurrence of two hydrogen bonds: (i) between the hydroxyl group of naringenin (O4 atom) and the nitrogen atom of the backbone of K103 (34.53%) and (ii) between the oxygen atom of naringenin (O2 atom) and the nitrogen atom of the backbone of G81 (17.6%) (Figure 7). Furthermore, we also calculated the interaction energy between all residues of the NS5 subunit that make contact with naringenin during MD simulation and found eighteen attractive interactions, with the most important being G79 and I145 (Figure 8). These results help to explain the high conformational stability of naringenin in the active site of NS5-methyltransferase, as well as the high affinity for this target. The carbon atoms of naringenin are shown in yellow.

Moreover, we also calculated the interaction energy between all residues of the NS5 subunit that made contact with naringenin during the MD simulation and found eighteen attractive interactions, the most important being G79 and I145 (Figure 8). These results help to explain the high conformational stability of naringenin in the active site of NS5-Methyltransferase, as well as the high affinity for this target.

## 5. Discussion

Flavonoids represent a source of active compounds against flaviviruses. This class of compounds possess a variety of pharmacological and biological properties. The antiviral activity of naringenin and 7-*O*-methyl-narigenin was previously demonstrated for different viruses, including hepatitis C, human rhinovirus 3, influenza B, DENV, ZIKV and chikungunya [55,56,57,58,59]. In addition, a recent study considered naringenin as a potential drug for the of treatment patients with COVID-19 [60]. These previous studies, as well as the present one, reinforce the therapeutic feature of each molecule. The promising results from the in silico and in vitro analyses performed here may elucidate the mechanism of action of the tested compounds. Additionally, our study highlights geopropolis as a valuable natural source for the isolation of flavonoids with antiviral potential.

In general, flavonoids are not highly toxic to cell lineages. Here, the cytotoxicity of subfractions and isolated flavonoids were compared to 6-MMPr, a thiopurine drug, already described as an anti-West Nile virus inhibitor [61]. Our group has also previously demonstrated the antiviral effects of this drug against ZIKV [33]. Due to the observed toxicity on the hexanic fraction, as well as the lack of significant compounds’ identification from the EtOH fraction, we decided to test only AcOEt-obtained flavonoids for all subsequent experiments. The hexanic toxic feature, on the other hand, can be beneficial when it comes to cancer cells lineages, such as HeLa and MCF-7 cells [62,63].

The flavonoids isolated from geopropolis, naringenin and 7-*O*-methyl-narigenin, demonstrated low cytotoxicity levels. This was also previously described for naringenin CC_50_ = 250 μM and CC_50_ = 87 µg/mL in in vitro analyses and clinical trials [4,55,64]. Meanwhile, 7-*O*-methyl-narigenin has already exhibited no toxicity toward Hela cells, yielding 100% cell viability at the tested concentrations [59]. The results herein reported, reinforce the potential profiles of these flavonoids as antiviral candidates.

The first stage of antiviral tests, the screening analysis, enabled a preview selection of compounds that indicated a therapeutic capacity could be used. Antiviral susceptibility tests seek to evaluate a series of decreasing concentrations, starting from the CC_20_ concentration, showing which concentration is compatible with the minimum capacity of antiviral action for each compound [65]. By observing the cytopathic effect, defined as a phenotypic analysis, in comparison with the mock control, it was possible to understand which extracts, fractions and substances isolated from geopropolis showed a significant reduction in viral-induced cytopathic effects [55,64,65].

In the second part of the antiviral tests, the viral titer on the supernatant was quantified by TCID_50_ after treatment with the extract, fractions and substances isolated from geopropolis. 6-MMPr was used as a positive control drug due to its previously demonstrated antiviral activity [33]. In addition to the quantification of viral infectious titers, the drug concentration required to induce cytotoxicity and antiviral activity was also measured. The SI (selective index) is the value assigned to the rate between CC_50_ and the value assigned to the title viral (IC_50_—inhibitory concentration). The higher the SI, the safer and, theoretically, more effective the drug is. Treatment with the tested compounds resulted in a significant reduction in viral titers for both ZIKV and DENV-2. The obtained data substantially justified the conclusions from the cytopathic effect reduction observed in the previously performed screening test. Other authors have already proved the antiviral activity of the flavonoid naringenin [4,55], which indicates that the variation molecule 7-*O*-methtyl-narigenin also has antiviral potential. The lack of information about this last substance highlights the importance of this study to support future analyses.

In addition to the in vitro studies, we performed an in silico analysis to better understand and compare the interactions between each substance and the main nonstructural proteins of DENV-2 and ZIKV: (i) NS3-helicase; (ii) NS3-protease; (iii) NS5-methyltransferase; and (iv) NS5-RdRp. First, we could not discern whether the tested compounds were active or not for the molecular targets using the scores obtained with Vina software. It is known that Vina can predict binding poses well; however, it is not able to rank active ligands in the top positions (most negative binding affinity). This can be explained by the fact that naringenin and 7-*O*-methyl naringenin have a very similar molecular structure [66]. After rescoring using a semi-empirical method (based on quantum mechanics), it was possible to quantify intermolecular interactions more accurately. Using this type of approach, it is possible to calculate the charge transfer events between the protein and the ligand, which is not possible in methods based on classical mechanics. Flavonoids seem to principally impair the activity of proteins related to replication, such as the NS2B-NS3 and NS5-RdRp complex of dengue and ZIKV [3,67]. According to the obtained molecular modeling data, naringenin is able to closely interact with NS5-methyltransferase of DENV-2 with higher affinity, ΔH _bind_ = −18.13. Additionally, the molecular dynamics simulation complemented and supported the high conformational stability of naringenin in the active site of NS5-methyltransferase through the analysis of hydrogen bonds. Considering this, our in silico results match with the post-treatment experimental data, which suggest that naringenin plays an important role in the DENV replication process through the Mtase domain, therefore being related to the activity inhibition of these virus proteins. [68]. These proteins are essential in the viral life cycle, and their inhibition causes a deficiency in the production of viral particles. However, future experimental validation is needed to confirm this hypothesis.

## 6. Conclusions

Our data demonstrate that Jandaira geopropolis can serve as a valuable source of antivirals against urban epidemic flaviviruses. Specifically, we have identified that naringenin and 7-*O*-methyl naringenin, which are present in the flavonoid fraction of geopropolis, exhibit potent ZIKV and DENV antiviral effects. Despite the lower selectivity index of these flavonoids when compared to currently available antiviral drugs, it is possible to infer that these molecules are important lead compounds for the development of compelling and multitarget antiviral drugs against both ZIKV and DENV infections and possibly other viruses. Association studies of these compounds with antivirals such as 6-MMPr, used herein as a positive control, may also enhance the antiviral activity against Zika and dengue viruses. Further bioprospection of Jandaira geopropolis may also reveal as yet unidentified substances with pharmacological applications in several areas, including antivirals. Our bioinformatics analysis using docking and molecular dynamics simulations also shed light on the mechanism of action in naringenin and 7-*O*-methyl-narigenin. Taken together, our findings demonstrate that both flavonoids have potent antiviral effects against ZIKV and DENV and warrant further trials in animal models.

## Figures and Tables

**Table 1:** Accession codes for the protein structures used in the docking simulations.

Organism	PDB	Protein	Resolution (Å)	Reference
DENV-2	2BMF	NS3-helicase	2.41	[35]
	3U1I	NS3-protease	2.30	[36]
	2P41	NS5-methyltransferase	1.80	[37]
	5K5M	NS5-RdRp	2.00	[38]
ZIKV	5K8I	NS3-helicase	1.69	[39]
	5YOF	NS3-protease	1.51	[40]
	5WZ2	NS5-methyltransferase	2.60	[41]
	6LD2	NS5-RdRp	1.40	[42]

**Table 2:** Cytotoxicity of ethanolic extract (EtOH), ethyl acetate (AcOEt) and hexane fractions as well as naringenin and 7-*O-*methyl naringenin isolated from geopropolis.

Sample	Cytotoxicity ^a^	
	CC_50_ (µM/mL)	CC_20_ (µM/mL)
EtOH	2.47	0.641
AcOEt	2.29	0.293
Hexanic	10.30	18.76
Naringenin	241.6	86.43
7-*O*-methyl naringenin	240.4	105.2
Positive control (6-MMPr)	86.00	17.88

^a^ Cytotoxicity was determined using the MTT method in a culture of Vero cells.

**Table 3:** Cytotoxicity and antiviral effects against ZIKV upon treatment of Vero cells with naringenin, 7-*O*-methyl naringenin and 6-MMPr.

	^d#^ CC_20_ (μM/mL)	^a#^ CC_50_ (μM/mL)	^b#^ IC_50_ (μM/mL)	^c#^ SI
Naringenin	86.43	241.6	58.89	4.1
7-*O*-methyl naringenin	105.2	240.4	12.80	18.7
6-MMPr	60.5	291	24.5	11.9

^a^ CC_50_ (50% cytotoxic concentration) compound concentration to reduce 50% of viability. ^b^ IC_50_ (50% inhibitory concentration) compound concentration to reduce 50% viral titer when compared with untreated cells. ^c^ Selective index (SI) based on the ratio calculation of CC_50_ and IC_50_ values. ^d^ CC_20_ (20% cytotoxic concentration), the non-toxic concentration used in the antiviral assays. ^#^ Results are defined by the mean values of three independent experiments.

**Table 4:** Cytotoxicity and antiviral effects against DENV upon treatment of Vero cells with naringenin, 7-*O*-methyl naringenin and 6-MMPr.

	^d#^ CC_20_ (μM/mL)	^a#^ CC_50_ (μM/mL)	^b#^ IC_50_ (μM/mL)	^c#^ SI
Naringenin	86.43	241.6	42.91	5.6
7-*O*-methyl naringenin	105.2	240.4	30.95	7.7
6-MMPr	60.5	291	18.15	16.0

^a^ CC_50_ (50% cytotoxic concentration) compound concentration to reduce 50% of viability. ^b^ IC_50_ (50% inhibitory concentration) compound concentration to reduce 50% viral titer when compared with untreated cells. ^c^ Selective index (SI) based on the ratio calculation of CC50 and IC50 values. ^d^ CC_20_ (20% cytotoxic concentration), the non-toxic concentration used in the antiviral assays. ^#^ Results are defined by the mean values of three independent experiments.

## Data Availability

The produced experimental data from the findings in this study will be available to interested investigators.

## Supplementary Materials

The following supporting information can be downloaded at: https://www.mdpi.com/article/10.3390/pharmaceutics15102494/s1, Figure S1: Chemical structure of the two tested flavonoids, naringenin (A) and (B) 7-*O*-methyl naringenin; Figure S2: Docking scores obtained for the poses predicted by the Vina software.
