# Supplementary material for: Antiviral Activity of Flavonoids from Geopropolis of the Brazilian Jandaira Bee against Zika and Dengue Viruses
Source: Pharmaceutics. 2023 Oct 19;15(10):2494. doi: 10.3390/pharmaceutics15102494 (PMC10609720; doi:10.3390/pharmaceutics15102494)
Supplement: Supplementary file 1 [file pharmaceutics-15-02494-s001.zip › pharmaceutics-2459298-supplementary.pdf]

SUPPLEMENTARY MATERIAL

Antiviral Activity of flavonoids from Geopropolis of the Brazilian Jandaira bee against Zika and dengue viruses

Poliana Gomes da Silva[1], Elton José Ferreira Chaves[1], Tania Maria Sarmento Silva[2], Gerd Bruno Rocha[3], Willyenne Marília Dantas[1,4], Ronaldo Nascimento de Oliveira[4] and Lindomar José Pena[1*].

[1] Laboratory of Virology and Experimental Therapy (LaviteLE) - Department of Virology, Aggeu Magalhães Institute (IAM), Oswaldo Cruz Foundation (Fiocruz), Recife, Pernambuco, Brazil; poligs250@gmail.com; chavesejf@cbiotec.ufpb.br

[2] Phytochemical Bioprospecting Laboratory, Department of Chemistry, Federal Rural University of Pernambuco, Recife, Pernambuco, Brazil; sarmentosilva@gmail.com

[3] Laboratory of Computational Quantum Chemistry, Department of Chemistry, Federal University of Paraiba, João Pessoa, Paraiba, Brazil. gbr@quimica.ufpb.br

[4] Bioactive Compounds Synthesis Laboratory, Department of Chemistry, Federal Rural University of Pernambuco (UFRPE), Recife, Brazil ronaldon38@gmail.com; dantaswillyenne@gmail.com

* Correspondence: lindomar.pena@focruz.br; lindomarfocruz@gmail.com

Keywords:; *Zika virus; dengue, antivirals; Naringenin; 7-O-methyl naringenin; Geopropolis; Molecular simulations.*

(A) (B)

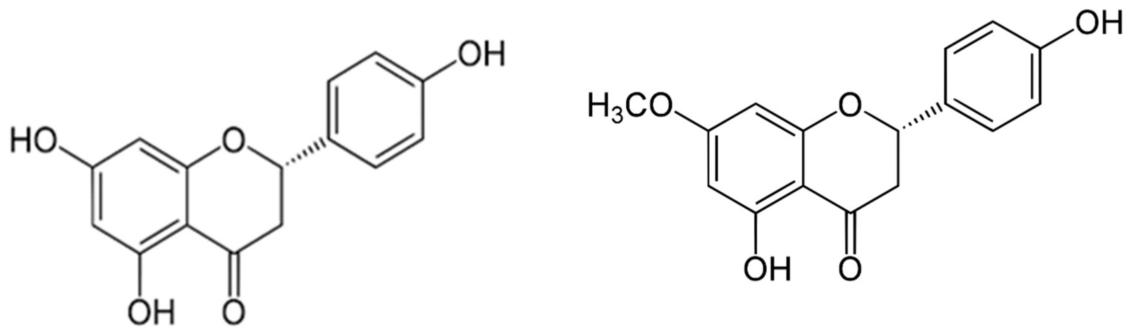

Figure S1. Chemical structure of the two tested flavonoids. Naringenin (A) and (B) 7-*O*-methyl naringenin.

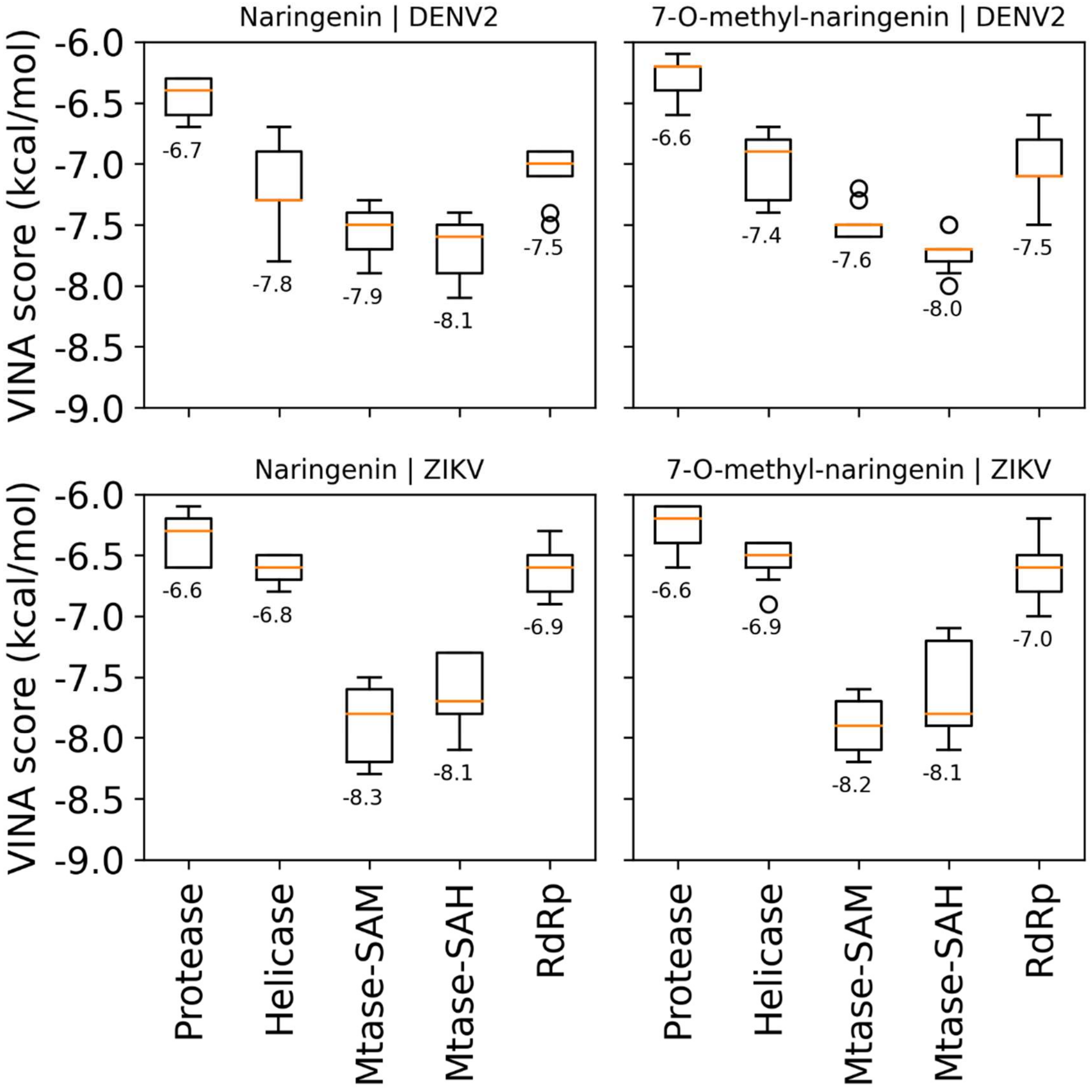

Figure S2. Docking scores obtained for the poses predicted by the Vina software.
